# Epigallocatechin-3-gallate inhibits the growth and increases the apoptosis of human thyroid carcinoma cells through suppression of EGFR/RAS/RAF/MEK/ERK signaling pathway

**DOI:** 10.1186/s12935-019-0762-9

**Published:** 2019-02-28

**Authors:** Dongdong Wu, Zhengguo Liu, Jianmei Li, Qianqian Zhang, Peiyu Zhong, Tieshan Teng, Mingliang Chen, Zhongwen Xie, Ailing Ji, Yanzhang Li

**Affiliations:** 1 School of Basic Medical Sciences, Henan University College of Medicine, Kaifeng, 475004 Henan China; 2 State Key Laboratory of Tea Plant Biology and Utilization, Anhui Agricultural University, 130 Changjiang West Road, Hefei, 230036 Anhui China; 3 Henan International Joint Laboratory for Nuclear Protein Regulation, Henan University, Kaifeng, 475004 Henan China

**Keywords:** EGCG, Thyroid carcinoma, Apoptosis, Angiogenesis, Signaling pathway

## Abstract

**Background:**

Thyroid cancer is the most common type of endocrine malignancy and the incidence rate is rapidly increasing worldwide. Epigallocatechin-3-gallate (EGCG) could suppress cancer growth and induce apoptosis in many types of cancer cells. However, the mechanism of action of EGCG on the growth of human thyroid carcinoma cells has not been fully illuminated.

**Methods:**

Cell proliferation and viability were detected by EdU and MTS assays. Cell cycle distribution was measured by flow cytometry. Migration and invasion were evaluated by scratch and transwell assays. Apoptotic levels were detected by TUNEL staining and western blotting. The protein levels of EGFR/RAS/RAF/MEK/ERK signaling pathway were detected by western blotting. The in vivo results were determined by tumor xenografts in nude mice. The in vivo proliferation, tumor microvessel density, and apoptosis were detected by immunohistochemistry.

**Results:**

EGCG inhibited the proliferation, viability, and cell cycle progression in human thyroid carcinoma cells. EGCG decreased the migration and invasion, but increased the apoptosis of human thyroid carcinoma cells. EGCG reduced the protein levels of phospho (p)-epidermal growth factor receptor (EGFR), H-RAS, p-RAF, p-MEK1/2, and p-extracellular signal-regulated protein kinase 1/2 (ERK1/2) in human thyroid carcinoma cells. EGCG inhibited the growth of human thyroid carcinoma xenografts by inducing apoptosis and down-regulating angiogenesis.

**Conclusions:**

EGCG could reduce the growth and increase the apoptosis of human thyroid carcinoma cells through suppressing the EGFR/RAS/RAF/MEK/ERK signaling pathway. EGCG can be developed as an effective therapeutic agent for the treatment of thyroid cancer.

## Background

Thyroid cancer is the most common endocrine malignancy with increasing incidence in recent years [1, 2]. Thyroid cancer of follicular cell origin accounts for the majority of thyroid malignancies, with other cancers deriving from parafollicular cells, namely medullary thyroid cancer (MTC) [3]. Almost 50% of patients with MTC present with lymph node metastases and 10% with distant metastatic disease [4]. Follicular cell-derived cancers include differentiated follicular thyroid cancer (FTC) and papillary thyroid cancer (PTC), as well as the undifferentiated anaplastic thyroid cancer (ATC). FTC and PTC can further progress to poorly differentiated thyroid cancer [5–7]. ATC is less frequent and has a median survival time of less than 5 months [8]. Currently, there are no available therapies in the treatment of aggressive thyroid carcinomas, which is partly attributed to the loss of capacity to uptake iodine [5, 9, 10]. Thus, it is an urgent need to develop novel agents/drugs for the treatment of thyroid carcinoma.

Green tea is a popular beverage and has received considerable attention worldwide because of its beneficial effects on human health [11–13]. The health benefits of green tea are mainly attributed to catechins, including catechin, epicatechin, epigallocatechin, epigallocatechin-3-gallate (EGCG), and epicatechin-3-gallate [14, 15]. EGCG, the most bioactive and abundant catechin in green tea, has shown therapeutic effects against several diseases, such as cancer, obesity, cardiovascular disease, metabolic syndrome, and neurodegenerative disease [15–18]. EGCG can modulate multiple cellular signaling and metabolic pathways including inhibition of cancer cell growth, metastasis, invasion, and induction of apoptosis in different cancer cells and animal models [15, 16, 19–21]. In addition, EGCG selectively decreases cell growth and increases apoptosis in cancer cells without adversely affecting normal cells [22, 23]. However, the mechanism of action of EGCG on the growth of thyroid carcinoma has not been fully illuminated.

In the present study, we examined the effect and mechanism of EGCG on the proliferation, migration, invasion, cell cycle, and apoptosis of human thyroid carcinoma cells in vitro. We further determined the effects of EGCG on tumor growth, apoptosis, and angiogenesis in nude mice bearing human thyroid carcinoma xenografts.

## Materials and methods

### Cell culture

Human thyroid carcinoma cell lines TT, TPC-1, and ARO were purchased from CoBioer Biosciences Co., Ltd. (Nanjing, Jiangsu, China). All cell lines were maintained in RPMI1640 media supplemented with 10% fetal bovine serum (FBS), 100 µg/ml streptomycin, and 100 U/ml penicillin. Cell culture was kept in an incubator with 5% CO_2_ at 37 °C. Confluent cells were maintained in serum-free RPMI1640 media for overnight starvation. Then the cells were respectively treated with 10, 25, 50, 100, and 200 µM EGCG. The control group was treated with phosphate-buffered saline (PBS). After 24 h of treatment, the cells were used in subsequent in vitro experiments.

### Cell growth assay

Cell proliferation was assessed by the Cell-Light EdU Apollo 567 In Vitro Imaging Kit (RiboBio, Guangzhou, Guangdong, China) following the manufacturer’s instructions. Cell proliferation rate = (number of EdU-positive cells)/(total number of cells) × 100% [24]. Cell viability was determined by the CellTiter 96 AQ_ueous_ One Solution Cell Proliferation Assay kit (MTS; Promega, Madison, WI, USA) according to the manufacturer’s recommended protocols. Cell viability was calculated as a percentage of the untreated control cells.

### Wound healing assay

Confluent cells were scratched using a sterile pipette tip to create a wound. Then the detached cells were removed by washing twice with PBS. The migration distance was observed under an Olympus CKX41 microscope and measured using Image J software (National Institute for Health, Bethesda, MD, USA). The migration rate (MR) was calculated as MR (%) = [(A − B)/A] × 100, where A is the width at 0 h, and B is the width at 24 h [25].

### Migration and invasion assays

1 × 10^5^ cells were seeded into the upper chamber in serum-free RPMI 1640 medium uncoated or coated with Matrigel (BD Biosciences, San Jose, CA, USA). Then 500 µl corresponding medium containing 10% FBS was added into the lower chamber. After incubation for 24 h, the cells were scrubbed with a cotton tip swab. The cells on the bottom surface of the membrane were fixed with 4% paraformaldehyde for 20 min at 37 °C and stained with 0.1% crystal violet for 10 min at 37 °C. Cell number was counted with a Zeiss Axioskop 2 plus microscope (Carl Zeiss, Thornwood, NY, USA).

### TdT-mediated dUTP-biotin nick end labeling (TUNEL) assay

TUNEL assay was conducted using an In Situ Cell Death Detection Kit (Beyotime Biotechnology, Shanghai, China) according to the manufacturer’s instructions. The stained cells were observed under a fluorescent microscope (Eclipse Ti, Nikon, Melville, NY, USA). The percentage of positive cells was calculated using Image J software.

### Cell cycle analysis

Cells (1 × 10^6^ cells per well) were seeded in 6-well plates and incubated for 24 h. Cells were collected and fixed in 1 ml of 70% ice-cold ethanol, incubated at 4 °C for 2 h, and centrifuged at 1000×*g* for 5 min to remove the ethanol. Cellular pellets were washed with PBS and suspended in 0.5 ml of PBS containing 50 µg/ml RNase A for 30 min at 37 °C. Then propidium iodide (50 µg/ml) staining solution was added, and cells were incubated for 30 min at 37 °C in the dark. The samples were measured by flow cytometry to determine the cell cycle distribution.

### Western blotting

Total protein was extracted from TT, TPC-1, and ARO cells. Western blotting was employed to detect the expression of target proteins. The primary antibodies, including anti-epidermal growth factor receptor (EGFR), anti-phospho (p)-EGFR, anti-H-RAS, anti-RAF, anti-p-RAF (Ser259), anti-MEK1/2, anti-p-MEK1/2 (Ser217/221), anti-extracellular signal-regulated protein kinase 1/2 (ERK1/2), and anti-p-ERK1/2 (Thr202/Tyr204) antibodies were purchased from Cell Signaling Technology (CST, Danvers, MA, USA). Anti-B-cell lymphoma-2 (Bcl-2), anti-Bcl-2-associated X protein (Bax), anti-cleaved caspase-3 (cas-3), anti-cleaved poly adenosine diphosphate-ribose polymerase (PARP), and anti-β-actin antibodies were purchased from ProteinTech (Chicago, IL, USA). The horseradish peroxidase-conjugated secondary antibodies were purchased from CST. The results were normalized to the expression level of β-actin. The proteins were visualized using an enhanced chemiluminescence system (Thermo Fisher Scientific, Rockford, IL, USA). The bands were semi-quantified using Image J software.

### Animal study

Animal experiments were approved by the Committee of Medical Ethics and Welfare for Experimental Animals of Henan University School of Medicine (HUSOM-2017-207) in compliance with the Experimental Animal Regulations formulated by the National Science and Technology Commission, China. Animal studies were performed as previously described with slight modifications [26]. Thirty-six BALB/C nude mice (4-week-old, male) were purchased from Beijing Vital River Laboratory Animal Technology Co., Ltd. (Certificate No. SCXK (Jing) 2011-0011, Beijing, China). TT, TPC-1, and ARO cells (2 × 10^6^ cells in 200 µl PBS) were subcutaneously inoculated into the right flanks of mice. At 24 h after inoculation, the mice were randomly divided into six groups (n = 6 per group). EGCG (10, 25, 50, 100, and 200 µM) was continuously administered subcutaneously (near the implanted tumor) for 28 days. The control group was treated with PBS. Body weighs and tumor volumes were measured daily during the experiment. The tumor volumes were determined as volume = L × W^2^/2, where L is the longest dimension parallel to the skin surface and W is the dimension perpendicular to L and parallel to the surface [27]. At the end of the experiment, mice were sacrificed and tumors were weighted. The tumor inhibition rate (IR) was calculated as IR (%) = [(A − B)/A] × 100, where A is the average tumor weight of the control group, and B is that of the treatment group [26].

### Hematoxylin and eosin (HE) staining

Tumor specimens were fixed in 10% neutral buffered formalin and embedded in paraffin. Sections were cut at a thickness of 5 µm and then stained with HE. Tumor tissues were observed under a Zeiss Axioskop 2 plus microscope.

### Immunohistochemistry (IHC) and evaluation

Tumor tissues were stained with anti-Ki67 antibody (CST, Danvers, MA, USA). Ki67-positive cells were observed and photographed with a Zeiss Axioskop 2 plus microscope. The proliferation index (PI) was quantified by determining the number of Ki67 positive cells among the total number of resting cells [28]. Cluster of differentiation 31 (CD31) is an important biomarker for vascular endothelial cells, and its immunostaining density is considered the tumor microvessel density (MVD) [29]. Tumor tissues were stained with anti-CD31 antibody (CST, Danvers, MA, USA) to detect the tumor MVD. Vessels with a clearly defined lumen or well-defined linear vessel shape were counted from the representative tumor zone using a Zeiss Axioskop 2 plus microscope. Then tumor tissues were stained with anti-cleaved PARP antibody (ProteinTech, Chicago, IL, USA) and anti-p-ERK1/2 antibody (CST, Danvers, MA, USA) respectively. Positive cells were observed and photographed with a Zeiss Axioskop 2 plus microscope. The percentage of positive staining cells was measured by determining the number of positive cells among the total number of cells.

### Statistical analysis

Data are presented as mean ± standard error of the mean (SEM). The differences between multiple groups were analyzed by one-way analysis of variance using SPSS 17.0 software, followed by Tukey’s test. A *P* value of less than 0.05 was considered to be statistically significant.

## Results

### EGCG inhibits the proliferation, viability, and cell cycle progression in human thyroid carcinoma cells

**Fig. 1:**
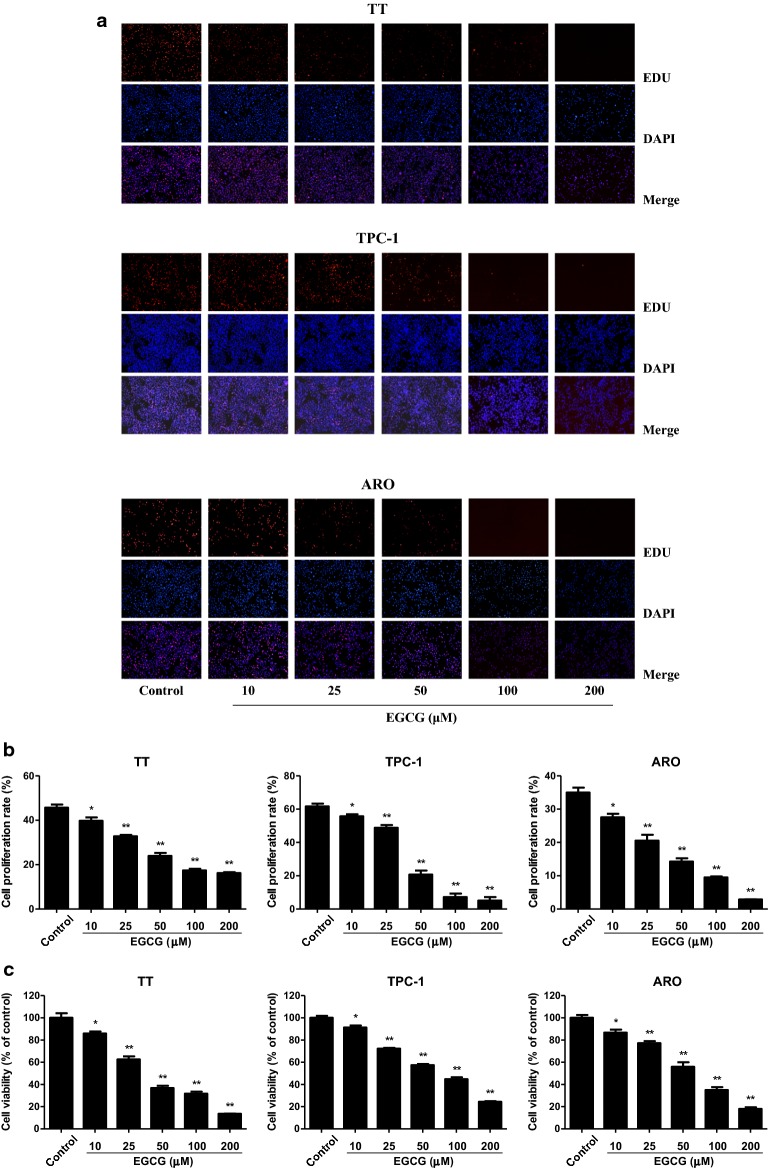
Effects of EGCG on the proliferation and viability of human thyroid carcinoma cells. **a** DNA replication activities of TT, TPC-1, and ARO cells in each group were examined by EdU assay; original magnification ×100. **b** The proliferation rate of each group was analyzed. **c** The percentages of viable cells were determined using MTS assay and the cell viability of the control group was taken as 100%. Data are presented as mean ± SEM of three independent experiments. *P < 0.05, **P < 0.01 compared with the control group

**Fig. 2:**
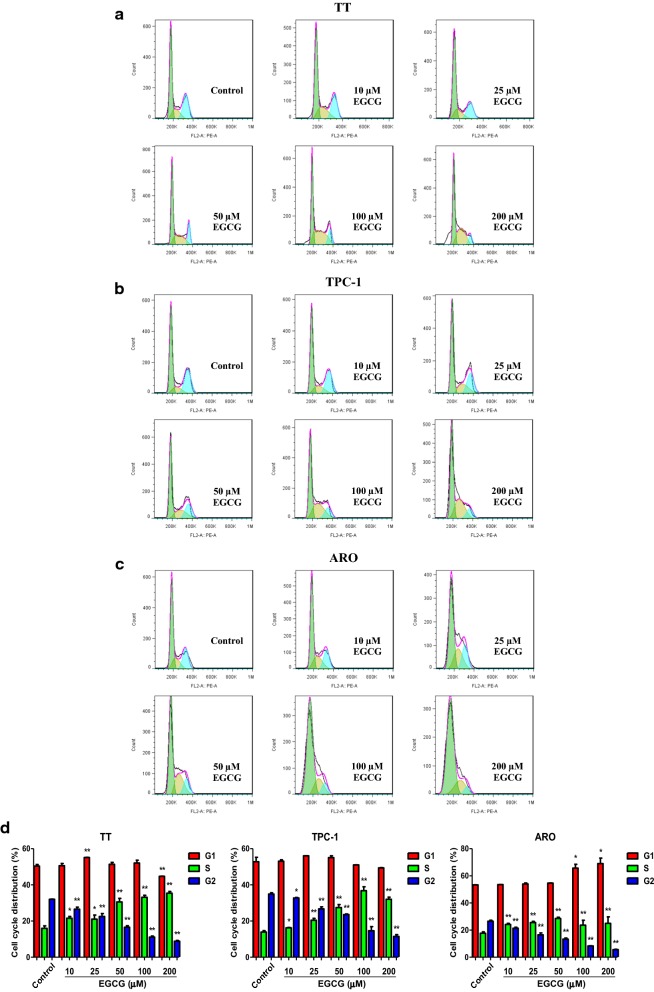
Effects of EGCG on cell cycle progression in human thyroid carcinoma cells. **a** Flow cytometry assay was used to determine cell cycle distribution in TT, TPC-1, and ARO cells. **b** Cell cycle distribution was analyzed. Data are presented as mean ± SEM of three independent experiments. *P < 0.05, **P < 0.01 compared with the control group

As shown in Fig. 1, the proliferation and viability of TT, TPC-1, and ARO cells were inhibited by 10–200 µM EGCG in a dose-dependent manner. Cell cycle progression is involved in cancer cell proliferation [30, 31]. Our results showed that 10–200 µM EGCG increased the proportion of cells entering the S phase and decreased the proportion of cells entering the G2 phase (Fig. 2), indicating that EGCG could induce cell cycle arrest at S phase in human thyroid carcinoma cells.

### EGCG decreases the migration and invasion of human thyroid carcinoma cells

**Fig. 3:**
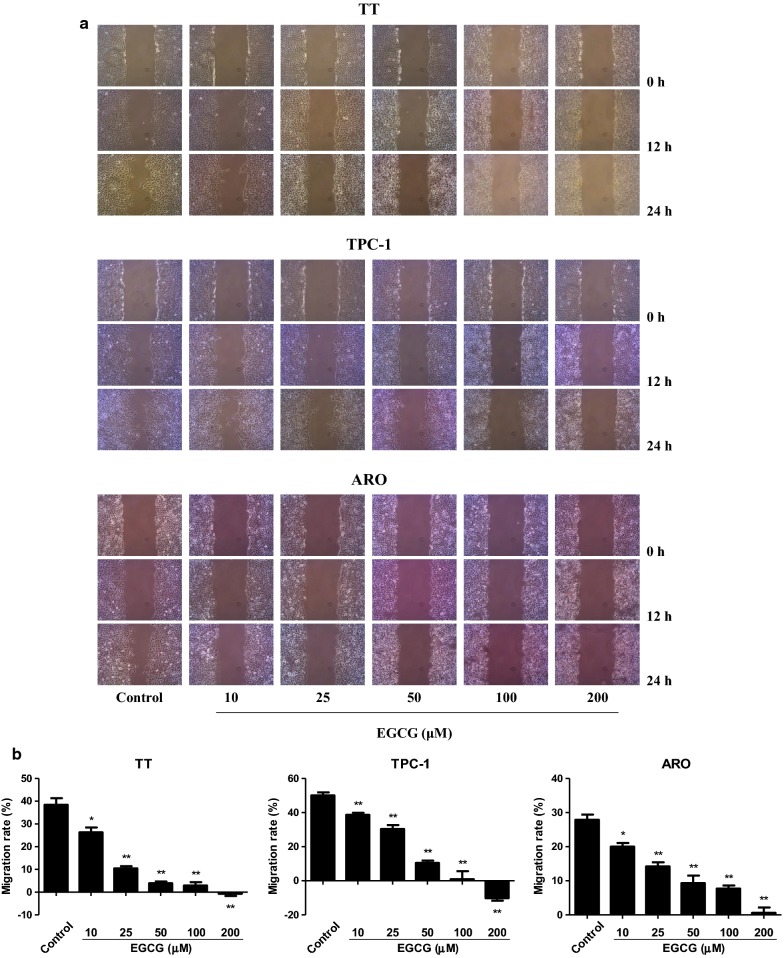
Effects of EGCG on the migration of human thyroid carcinoma cells. **a** The effect of EGCG on cell migration was measured by wound healing assay; original magnification ×100. **b** The migration rates of TT, TPC-1, and ARO cells were calculated by the formula shown above. Data are presented as mean ± SEM of three independent experiments. *P < 0.05, **P < 0.01 compared with the control group

**Fig. 4:**
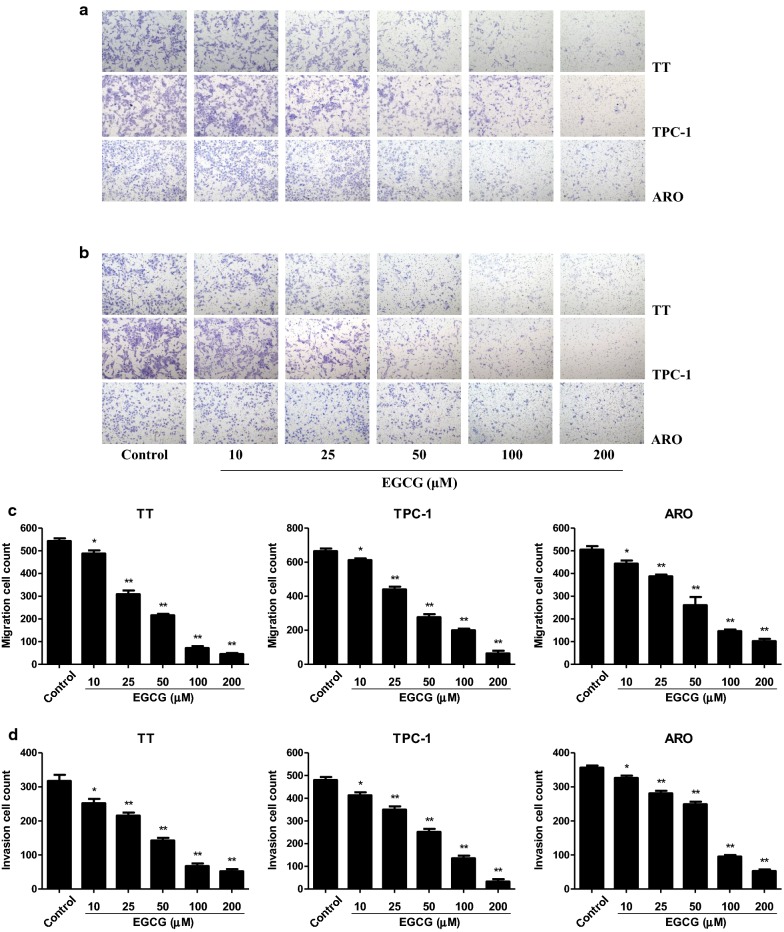
Effects of EGCG on the migration and invasion of human thyroid carcinoma cells. **a** Transwell assay was performed to assess the migration of TT, TPC-1, and ARO cells; original magnification ×200. **b** Transwell assay was performed to assess the invasion of TT, TPC-1, and ARO cells; original magnification ×200. **c** The numbers of the migrated cells were calculated. **d** The numbers of the invasive cells were calculated. Data are presented as mean ± SEM of three independent experiments. *P < 0.05, **P < 0.01 compared with the control group

In scratch migration assay, 10–200 µM EGCG dose-dependently decreased the migration capabilities of human thyroid carcinoma cells (Fig. 3). Transwell analysis showed that 10–200 µM EGCG increasingly inhibited the migration and invasion capacities of human thyroid carcinoma cells (Fig. 4). These results together suggest that EGCG could decrease the migration and invasion of human thyroid carcinoma cells.

### EGCG increases apoptosis of human thyroid carcinoma cells

**Fig. 5:**
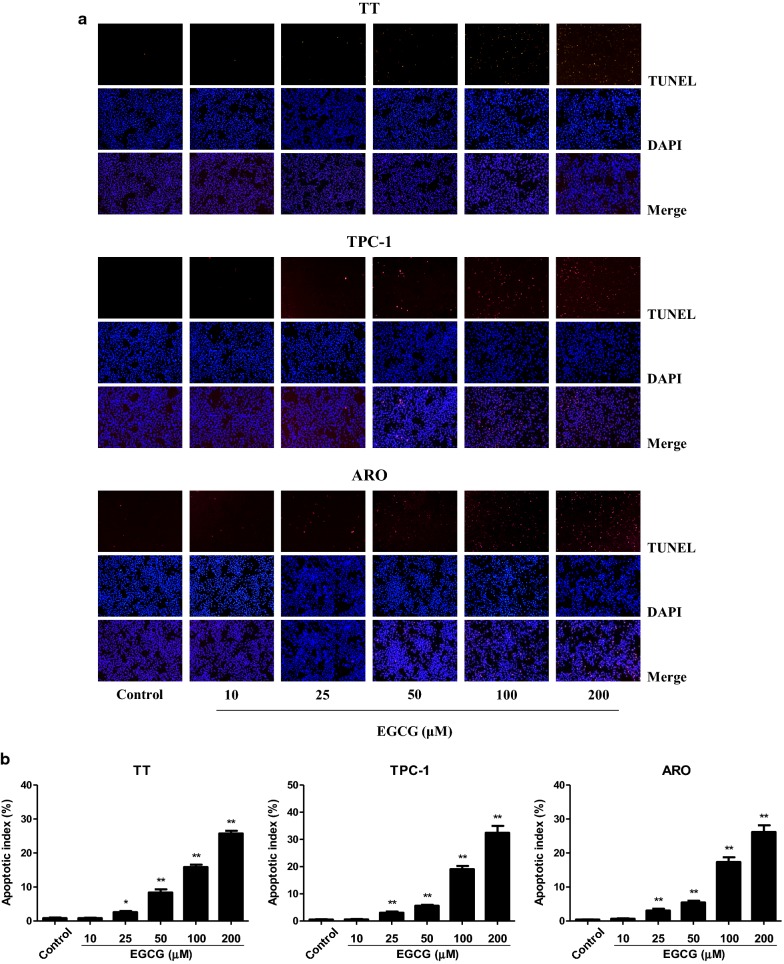
Effects of EGCG on the apoptosis of human thyroid carcinoma cells. **a** The apoptotic levels of TT, TPC-1, and ARO cells were measured by TUNEL staining; original magnification ×100. **b** The apoptotic index was calculated by the formula: the apoptotic index = (positively stained apoptotic cells)/(total number of cells) × 100%. Data are presented as mean ± SEM of three independent experiments. *P < 0.05, **P < 0.01 compared with the control group

**Fig. 6:**
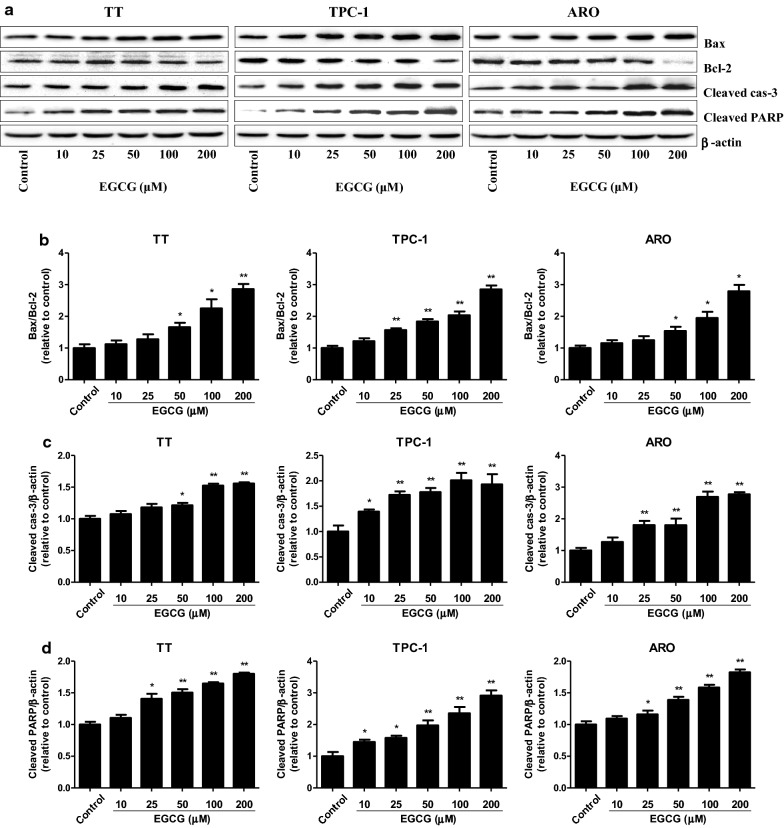
Effects of EGCG on the protein levels of Bax, Bcl-2, cleaved cas-3, and cleaved PARP in human thyroid carcinoma cells. **a** Western blotting analysis for the expression levels of Bax, Bcl-2, cleaved cas-3, and cleaved PARP in TT, TPC-1, and ARO cells. β-actin was used as the loading control. **b** The expression ratio of Bax/Bcl-2 was quantified. **c** The expression level of cleaved cas-3 was quantified. **d** The expression level of cleaved PARP was quantified. Data are presented as mean ± SEM of three independent experiments. *P < 0.05, **P < 0.01 compared with the control group

As shown in Fig. 5, 25–200 µM EGCG increased the apoptotic index in TT, TPC-1, and ARO cells in a dose-dependent manner. The ratio between Bax and Bcl-2 is an important factor in the regulation of apoptosis. Increased Bax/Bcl-2 ratio is a normal phenomenon in mitochondrial-mediated apoptosis in mammalian cells [32, 33]. As shown in Fig. 6a, b, the Bax/Bcl-2 ratio was increased by 50–200 µM EGCG. The expression levels of cleaved cas-3 and cleaved PARP in human thyroid carcinoma cells showed similar trends (Fig. 6a, c, d). These results show that EGCG could induce mitochondrial-mediated apoptosis in human thyroid carcinoma cells.

### EGCG suppresses the EGFR/RAS/RAF/MEK/ERK signaling pathway in human thyroid carcinoma cells

**Fig. 7:**
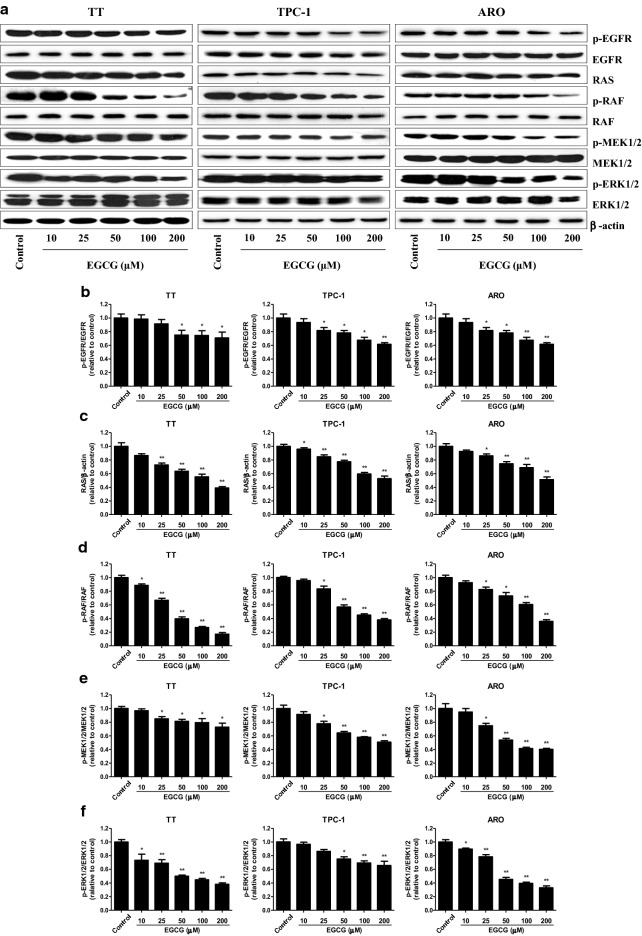
Effects of EGCG on the EGFR/RAS/RAF/MEK/ERK signaling pathway in human thyroid carcinoma cells. **a** Western blotting analysis of the expression levels of p-EGFR, EGFR, RAS, p-RAF, RAF, p-MEK1/2, MEK1/2, p-ERK1/2, and ERK1/2 in TT, TPC-1, and ARO cells. β-actin was used as the loading control. **b**–**f** The intensities of the bands were quantified by densitometry analyses and normalized by the amount of EGFR, β-actin, RAF, MEK1/2, and ERK1/2, respectively. Data are presented as mean ± SEM of three independent experiments. *P < 0.05, **P < 0.01 compared with the control group

The Ras/Raf/MEK/ERK cascade is a key signaling pathway that regulates many cellular functions including proliferation, survival, apoptosis, motility, differentiation, and metabolism [34–36]. Deregulation of the RAS/RAF/MEK/ERK pathway is regarded as a hallmark for driving tumorigenesis in a number of human cancers [37, 38]. Furthermore, it has been shown that RAS/RAF/MEK/ERK pathway is one of the downstream intracellular signals of EGFR [39]. As shown in Fig. 7, 50–200 µM EGCG gradually decreased the protein levels of p-EGFR, RAS, p-RAF, p-MEK1/2, and p-ERK1/2 in TT, TPC-1, and ARO cells. The results indicate that EGCG could suppress the EGFR/RAS/RAF/MEK/ERK pathway in human thyroid carcinoma cells.

### EGCG reduces the growth and angiogenesis and induces apoptosis of human thyroid carcinoma xenograft tumors in nude mice

**Fig. 8:**
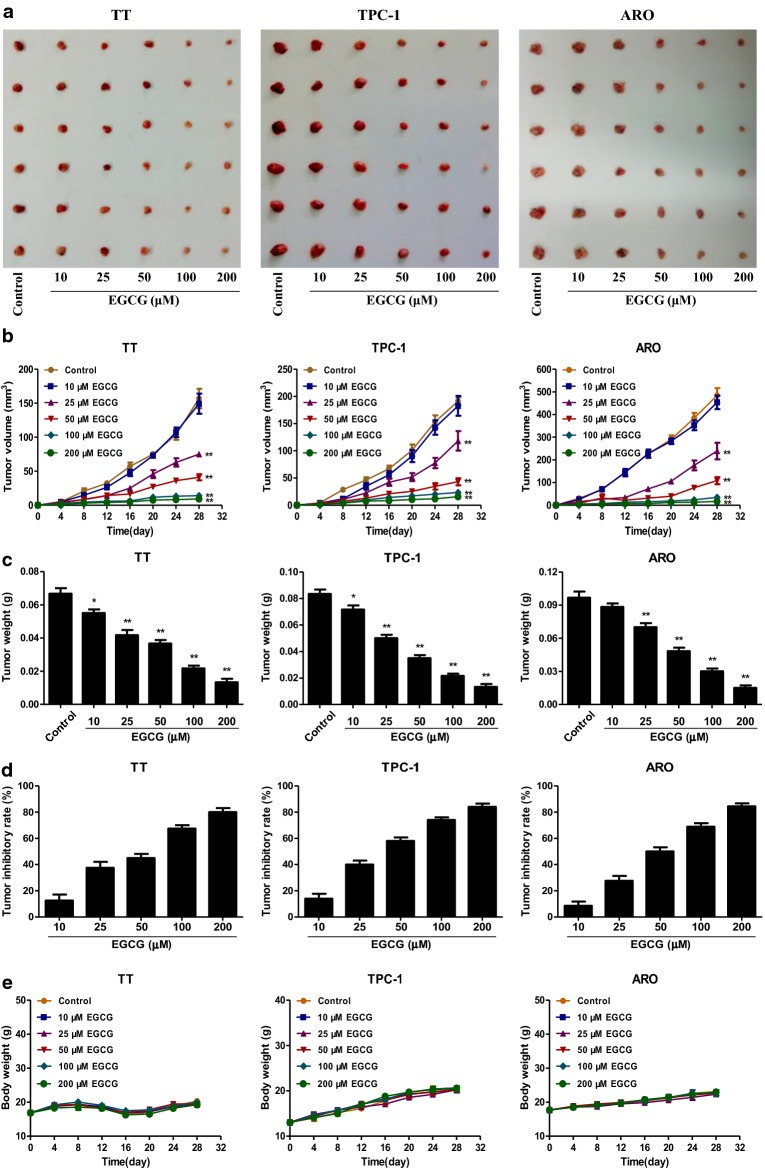
Effects of EGCG on the growth of TT, TPC-1, and ARO xenograft tumors in nude mice. **a** Representative xenografts dissected from different groups of nude mice were shown. **b** The tumor volume of each group was measured every day. **c**, **d** The tumors were weighed and the inhibition rates of tumor growth were calculated by the formula shown above. **e** The body weight change curve of each group during the experiment. Values are presented as mean ± SEM (n = 6). *P < 0.05, **P < 0.01 compared with the control group

**Fig. 9:**
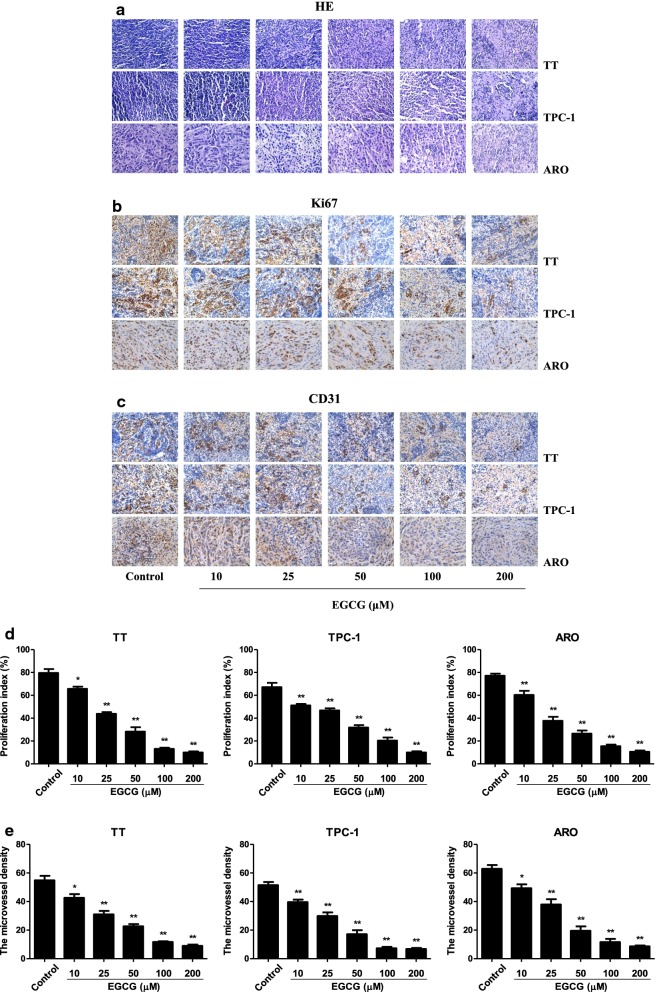
Effects of EGCG on the PI and MVD of human thyroid carcinoma xenografts. **a**–**c** Representive photographs of HE, Ki67, and CD31 staining in TT, TPC-1, and ARO xenograft tumors; original magnification ×400. **d**, **e** The PI and MVD were calculated by the formula shown above. Values are presented as mean ± SEM (n = 3). *P < 0.05, **P < 0.01 compared with the control group

**Fig. 10:**
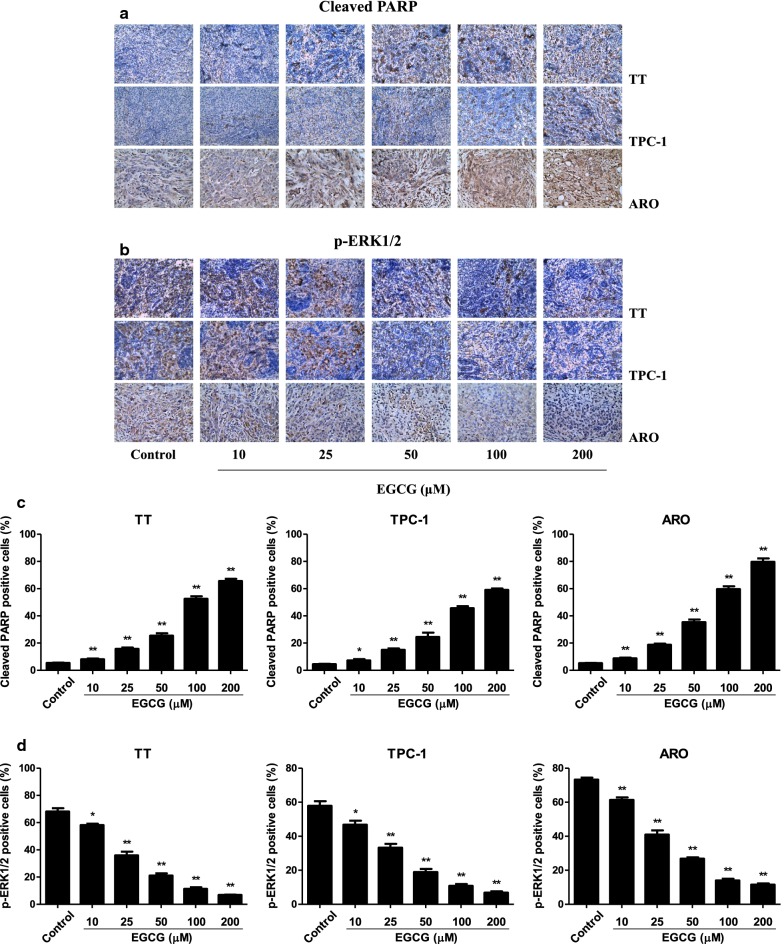
Effects of EGCG on the expression levels of cleaved PARP and p-ERK1/2 in human thyroid carcinoma xenografts. **a**, **b** Representive photographs of cleaved PARP and p-ERK1/2 staining in TT, TPC-1, and ARO xenograft tumors; original magnification ×400. **c**, **d** The cleaved PARP and p-ERK1/2 positive cells were calculated by the formula shown above. Values are presented as mean ± SEM (n = 3). *P < 0.05, **P < 0.01 compared with the control group

TT, TPC-1, and ARO cells have been widely used to establish mouse tumor models in cancer research [40–42]. Then we detected the effect of EGCG on the growth of human thyroid carcinoma xenograft in BALB/c nude mice. The results showed that treatment with 25–200 µM EGCG dose-dependently reduced the growth of xenograft tumors (Fig. 8a–d). However, no significant difference was observed in body weight between each group (Fig. 8e). IHC staining with Ki67 showed that the in vivo proliferation of human thyroid carcinoma cells was gradually decreased by treatment with 10–200 µM EGCG. The expression of CD31 in human thyroid carcinoma xenograft tumors exhibited a similar trend (Fig. 9). Furthermore, 10–200 µM EGCG dose-dependently increased the expression level of cleaved PARP, while a reverse trend was observed in the expression level of p-ERK1/2 (Fig. 10). In sum, these results demonstrate that EGCG could reduce the growth and angiogenesis, as well as induce apoptosis of human thyroid carcinoma xenograft tumors.

## Discussion

Thyroid cancer is the most common type of endocrine-related malignancy and its incidence rate is rapidly increasing worldwide [1, 2]. EGCG, the most abundant and bioactive catechin in green tea, can inhibit cancer cell growth, metastasis, invasion, and induce apoptosis in many types of cancer cells [15–21]. A previous study has shown that EGCG can inhibit the proliferation and motility of human thyroid papillary and follicular carcinoma cell lines [43]. However, the mechanism of action of EGCG on the growth of human thyroid carcinoma cells has not been fully illuminated. Human thyroid carcinoma cell lines TT, TPC-1, and ARO cells have been widely adopted in establishing tumor-bearing animal models [40–42]. In the present study, TT, TPC-1, and ARO cells were used to evaluate the effects of EGCG both in vitro and in vivo. The results indicated that treatment with 10–200 µM EGCG inhibited the proliferation, viability, cell cycle progression, as well as decreased the migration and invasion capabilities of TT, TPC-1, and ARO cells. In sum, these results reveal that EGCG could inhibit the proliferation, viability, migration, invasion, and cell cycle progression of human thyroid carcinoma cells.

Apoptosis, a form of programmed cell death, plays an essential role in the normal development and maintenance of cellular and tissue homeostasis in multicellular organisms [44, 45]. Two major apoptotic signaling pathways have been identified: an extrinsic pathway initiated by death receptors and an intrinsic pathway that occurs through the mitochondria [46]. The proteins of the Bcl-2 family are critical regulators of apoptosis in mammals, such as Bax and Bcl-2 [47]. Caspase-3 is activated by a variety of apoptotic stimuli and PARP can be cleaved by activated caspase-3, thus leading to the occurrence of apoptotic cascade [24, 26]. An increasing number of studies have suggested that EGCG could inhibit cellular proliferation and induce apoptosis in many types of cancer cells, including endometrial carcinoma, pheochromocytoma, chondrosarcoma, and B cell lymphoma [48–51]. Similarly, our results showed that EGCG dose-dependently increased the apoptotic index, protein levels of cleaved cas-3 and cleaved PARP, as well as the Bax/Bcl-2 ratio, suggesting the activation of mitochondria-mediated apoptotic pathway. The results indicate that EGCG induces mitochondrial-mediated apoptosis in human thyroid carcinoma cells. Furthermore, it has been reported that low dose radiation can induce senescence of human mesenchymal stromal cells, while the contribution to apoptosis is minimal [52]. Whether EGCG could induce senescence of human thyroid carcinoma cells needs to be further investigated.

RAS/RAF/MEK/ERK signaling pathway is involved in cancer development, maintenance, and progression [53, 54]. RAS/RAF/MEK/ERK pathway comprises the G-protein RAS and three dual-specific protein kinases RAF, MEK, and ERK. The binding of different ligands to receptor tyrosine kinases can induce the activation of RAS which in turn leads to the activation of RAF, MEK, and ERK [35, 55, 56]. RAS/RAF/MEK/ERK pathway has been reported to be one of the most frequently activated oncogenic signaling pathways in thyroid cancer [57]. In addition, recent studies have shown that RAS/RAF/MEK/ERK cascade is an important downstream intracellular signal of EGFR [39, 58]. A recent study indicates that forkhead box D3 is a tumor suppressor of colon cancer via the inhibition of EGFR/RAS/RAF/MEK/ERK signal pathway [59]. Another study has revealed that *Polygonatum odoratum* lectin induces apoptosis and autophagy by targeting EGFR-mediated RAS/RAF/MEK/ERK pathway in human MCF-7 breast cancer cells [60]. Similarly, our results showed that EGCG dose-dependently decreased the protein levels of p-EGFR, RAS, p-RAF, p-MEK1/2, and p-ERK1/2. The results suggest that EGCG is a tumor suppressor by increasing the apoptotic level in human thyroid carcinoma cells via EGFR/RAS/RAF/MEK/ERK signaling pathway.

A number of studies indicate that TT, TPC-1, and ARO cells have been widely adopted to establish subcutaneous xenograft models [40–42]. We therefore examined the effect of EGCG on the growth of human thyroid carcinoma xenograft tumors in BALB/c nude mice. The results showed that administration of EGCG reduced the growth of human thyroid carcinoma xenograft tumors in a dose-dependent manner. Ki67 is an important proliferative marker and has been widely used in detecting the proliferative activity of cancer cells [28, 61]. The results indicated that the expression levels of Ki67 in xenograft tumors were reduced by treatment with EGCG. CD31 has been considered an ideal biomarker for vascular endothelial cells and intratumoral MVD can be reflected by CD31 staining [29, 62]. Administration of EGCG decreased the expression level of CD31 in human thyroid carcinoma xenograft tumors. In addition, EGCG increased the expression level of cleaved PARP but decreased the expression level of p-ERK1/2. Taken together, these results suggest that EGCG could reduce the growth and angiogenesis, as well as induce apoptosis of human thyroid carcinoma xenograft tumors.

## Conclusions

In conclusion, our results demonstrate that EGCG could inhibit the growth and increase the apoptosis of human thyroid carcinoma cells via suppression of EGFR/RAS/RAF/MEK/ERK signaling pathway. EGCG can be developed as a novel therapeutic agent for the treatment of thyroid cancer.

## Abbreviations

MTC: medullary thyroid cancer
FTC: follicular thyroid cancer
PTC: papillary thyroid cancer
ATC: anaplastic thyroid cancer
EGCG: epigallocatechin-3-gallate
FBS: fetal bovine serum
PBS: phosphate-buffered saline
MR: migration rate
TUNEL: TdT-mediated dUTP-biotin nick end labeling
EGFR: epidermal growth factor receptor
p: phosphor
ERK1/2: extracellular signal-regulated protein kinase ½
Bcl-2: B cell lymphoma-2
Bax: Bcl-2-associated X protein
Cas-3: caspase-3
PARP: poly adenosine diphosphate-ribose polymerase
IR: inhibition rate
HE: hematoxylin and eosin
IHC: immunohistochemistry
PI: proliferation index
CD31: cluster of differentiation 31
MVD: microvessel density
SEM: standard error of the mean
